# Rare catastrophes and evolutionary legacies: human germline gene variants in *MLKL* and the necroptosis signalling pathway

**DOI:** 10.1042/BST20210517

**Published:** 2022-02-15

**Authors:** Sarah E. Garnish, Joanne M. Hildebrand

**Affiliations:** 1 The Walter and Eliza Hall Institute of Medical Research, Parkville, VIC 3052, Australia; 2 Department of Medical Biology, University of Melbourne, Parkville, VIC 3052, Australia

**Keywords:** loss of function gene variant, missense gene variant, MLKL, necroptosis, pathogenic mutation

## Abstract

Programmed cell death has long been characterised as a key player in the development of human disease. Necroptosis is a lytic form of programmed cell death that is universally mediated by the effector protein mixed lineage kinase domain-like (MLKL), a pseudokinase. MLKL's activating kinase, receptor interacting protein kinase 3 (RIPK3), is itself activated within context specific scaffolds of receptor interacting protein kinase 1 (RIPK1), Z-DNA Binding Protein-1 (ZBP1) or TIR domain-containing adaptor inducing interferon-β (TRIF). These core necroptosis modulating proteins have been comprehensively revealed as potent drivers and suppressors of disease in inbred mouse strains. However, their roles in human disease within the ‘real world’ of diverse genetic backgrounds, natural infection and environmental challenges remains less well understood. Over 20 unique disease-associated human germline gene variants in this core necroptotic machinery have been reported in the literature and human clinico-genetics databases like ClinVar to date. In this review, we provide an overview of these human gene variants, with an emphasis on those encoding MLKL. These experiments of nature have the potential to not only enrich our understanding of the basic biology of necroptosis, but offer important population level insights into which clinical indications stand to benefit most from necroptosis-targeted drugs.

## Introduction

**Figure 1.**
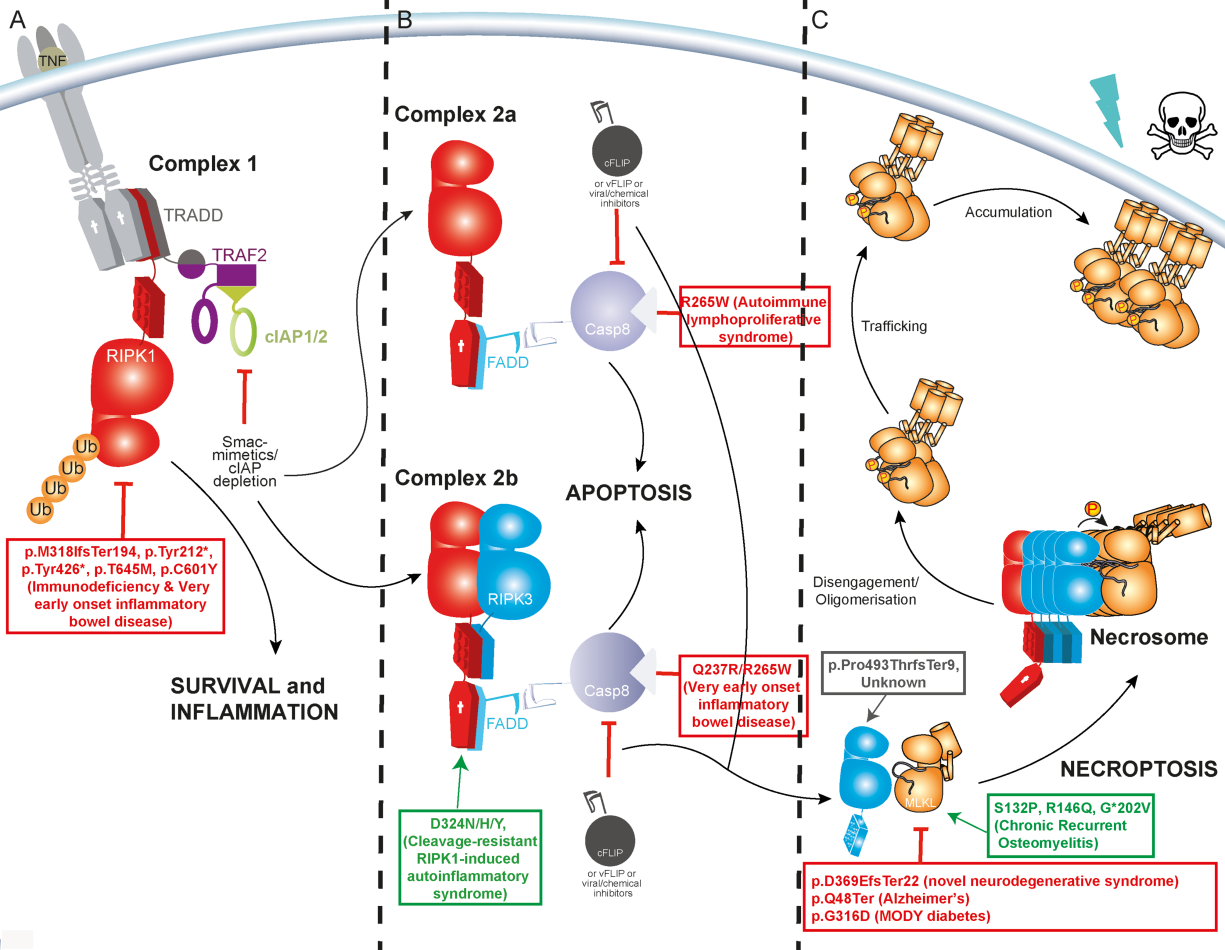
TNF-induced necroptosis occurs when upstream pro-survival and pro-apoptotic pathways are inhibited. (**A**) The binding of TNF to TNFR1 stimulates downstream nuclear factor-κB activation and other pro-survival, proinflammatory signals. (**B**) When cIAP1/2 activity is low, signalling is diverted to the formation of a death-induced signalling complex termed Complex II (a/b). This physically distinct complex is variably composed of TRADD, FADD, RIPK1, RIPK3 and the apoptosis initiator Caspase-8. (**C**) Necroptosis is activated when cellular conditions curb Caspase-8 activity and favour the assembly of RIPK1 and RIPK3, via their RIP Homotypic Interaction Motif (RHIM) domains, into a high molecular mass complex termed the necrosome. Here, RIPK3 is activated by autophosphorylation and MLKL is recruited and phosphorylated by RIPK3. MLKL dissociates from RIPK3 and oligomerised MLKL is trafficked to biological membrane [84,85]. The precise molecular events that lead to lytic permeabilization of the cell are still a matter of contention [86]. Sites of some selected published human germline mutations shown to be associated with human disease due to gain (red) or loss/reduction in function (green) are shown.

Programmed cell death takes many forms and is crucial to every aspect of normal animal development and homeostasis. The most well studied programmed cell death pathway is the caspase-*dependent* apoptosis. Apoptosis can be initiated by a range of intrinsic and extrinsic signals, and is commonly regarded as a ‘clean death’ characterised by the caspase-mediated disassembly of cells into highly phagocytosable, membrane enclosed bundles [1,2]. Unlike apoptosis, necroptosis is a caspase-*independent* form of programmed cell death that is characterised by the release of highly inflammatory cytokines, intracellular proteins, and nucleic acids into the extracellular space [3–6]. In turn, necroptosis is itself induced by inflammatory cytokines and danger- or pathogen-associated molecular patterns via their cognate transmembrane receptors or intracellular pattern recognition proteins like nucleic acid sensor Z-DNA Binding Protein-1. Of these various initiating stimuli, the most well studied route to necroptosis is downstream of tumour necrosis factor receptor 1 (TNFR1). This signal triggers the eventual formation of the RIPK1 and RIPK3-containing necrosome prior to terminal MLKL activation and cell death (Figure 1) [7–9].

Importantly, several additional, highly context-dependent facets to MLKL function have been reported (recently reviewed by Weir et al. [3]). These include roles in potassium channel mediated promotion of inflammasome activation [10], endocytic and exocytic modulation of cytokine release and membrane repair [11–14], myelin sheath remodelling [15], neutrophil extracellular trap formation [16,17] and even the direct suppression of intracellular bacteria [18,19].

**Figure 2.**
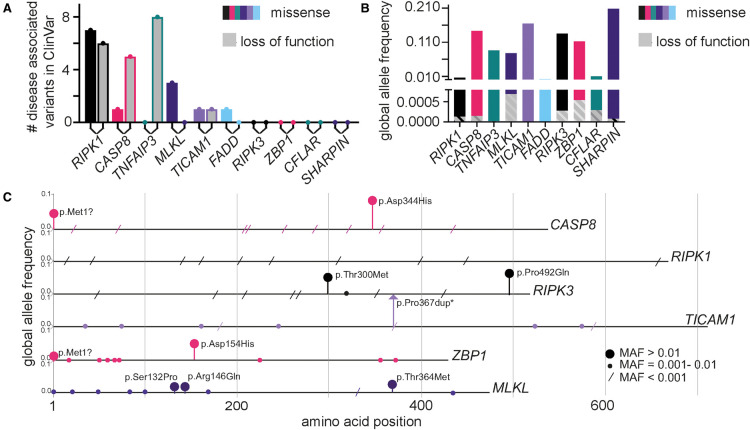
Frequency of gene variation and pathogenic gene variants in MLKL and upstream signalling modulators. (**A**) Total unique germline and *de novo* variants reported in ClinVar [52] as ‘pathogenic’, ‘likely pathogenic’, ‘associated’ or ‘risk factor. Somatic gene variants, contiguous copy-number variants, and gene variants not accompanied by a human disease condition were excluded from these counts. It is important to note that some patient mutations reported in the scientific literature may not have been submitted or updated by authors to the ClinVar database at the time of writing and thus are not included here. Disease causing variants in more upstream MLKL signalling modulators including death receptors, pattern recognition receptors, interferons and the NF-κB pathway are not presented here but are described in recent reviews [87–90]. (**B**) Summed global Minor Allele Frequency (MAF) of Missense and Loss of Function (LOF) variants as annotated by gnomAD at time of writing, (*n* = >280 000 alleles sequenced) [54]. Missense and LOF alleles flagged as ‘low confidence’ or ‘variant quality/annotation dubious’, alleles unique to non-canonical transcripts (with exception of CFLAR, where both long and short forms were included) or alleles with MAFs > 0.5 were excluded. (**C**) The top 10 missense variants used in calculating summed missense allele frequency in (**B**) and their global allele frequency plotted according to position in protein. *TICAM1 pPro367dup MAF = 0.3.

*Mlkl* gene knock-out and knock-in mutant mice have revealed necroptosis as a driver or protective factor in murine disease spanning every bodily system [20]. Simultaneously, massively parallel sequencing technologies have increased the efficiency and reduced the cost of human genotyping. This has led to the rapid accumulation of human genomic data and even paired phenotypic data in large open access databases like gnomAD [21] and the Global Biobank Engine [22]. Rare mutations in human *MLKL* have revealed neurological and metabolic pathologies where the *absence* of MLKL function plays a driving role [23–25], whilst rare and *de novo* patient mutations in MLKL signal modulators like *CASP8* and *RIPK1* [26], and common polymorphisms in *MLKL* [27] have provided insight into pathologies where *excessive* necroptotic signalling is an important contributor to inflammatory disease (Figure 2A). Together these studies of MLKL function in humans reveal some similarities, but also many key differences to observations made in inbred laboratory mouse strains housed under specific pathogen-free conditions [20].

## MLKL truncation/deletion mutations in human neurodegeneration

To date, only four individuals that are homozygous for a predicted loss of function (LOF) *MLKL* gene variant have been reported in the gnomAD database, a carefully curated collection of whole genome or exome data for over 140 000 human adults of diverse ancestry [21]. In 2020, Faergman et al. reported homozygosity of one of these *MLKL* variants (p.Asp369GlufsTer22, rs561839347) in two brothers diagnosed with progressive neurodegenerative spectrum disorder [24,28]. MLKL was not detectable by western blot in tissue derived from these patients, and patient-derived fibroblasts did not undergo programmed cell death when exposed to an exogenous necroptotic stimulus in culture*.* These data support gnomAD's ‘loss of function’ classification of p.Asp369GlufsTer22, making this the first peer-reviewed, clinical description of humans that are homozygous for an *MLKL* LOF gene variant. The brothers presented with an asymmetric lower limb weakness at the ages of 19 and 30, respectively, which developed into a novel progressive neurodegenerative disorder characterised by paresis, ataxia and dysarthria. Magnetic resonance imaging performed at 28 and 31 years, respectively, after onset of symptoms revealed severe global cerebral, cerebellar, cortical cerebellar fibers tract and brainstem atrophy and small, discrete periventricular T2-hyperintense white matter lesions. All pathology was limited to the nervous system and the patients did not exhibit any increased susceptibility to infections. It is important to note that this *MLKL* mutation also segregated with a homozygous in-frame deletion of one amino acid in the adjacent *FA2H* gene (fatty acid 2-hydroxylase), and a maternally inherited missense amino acid substitution in the X-linked gene *AP1S2* (vesicle sorting and transport) in these patients. While *FA2H* variant function was not impaired when examined in vitro and the *AP1S2* variant is classified as ‘benign’ based on ACMG guidelines [29], the influence of these and other genetic/environmental modifiers cannot be fully excluded from the severe phenotype exhibited by these individuals.

Another *MLKL* LOF gene variant (p.Gln48Ter, rs763812068) was found to be >20 fold enriched in a cohort of Hong Kong Chinese patients suffering from the neurodegenerative disease Alzheimer's, relative to a large ancestry-matched population [23]. This *MLKL* LOF variant is specific to populations of Southern Chinese descent, was only present in heterozygotes, and was not linked to any predicted damaging mutations in the adjacent *FA2H* gene. Wang et al. reported that shRNA-mediated knock down of *MLKL* in APPswe-293 cells led to an increased ratio of amyloid beta proteins Aβ42 to Aβ40. Further study of the precise role of MLKL in modulating levels of these proteins in neuronal cells remains an important area of future investigation.

Homozygous and heterozygous *Mlkl* gene knock-out (*Mlkl^KO/KO^*) mice do not appear to exhibit the spontaneous, progressive neurodegeneration observed in the patients described by Faergman et al. [24] and Wang et al. [23], implicating the contribution of additional genetic and/or environmental modifiers or key-interspecies differences in MLKL function. MLKL protein is present at low to undetectable levels in the healthy adult mouse brain and spinal cord [30–32], and was reported to be dispensable to the onset and severity of mouse autoimmune lateral sclerosis (ALS) [32]. There are reports however that MLKL is induced following injury to the mouse cerebral cortex and spinal cord [33,34]. *Mlkl^KO/KO^* mice also exhibit a reduced propensity for demyelination in several other independent experimental mouse disease models. This manifests as improved outcomes in mouse models of Parkinson's disease [35] and multiple sclerosis [36], and poorer outcomes in models of physical nerve injury [15]. These mouse data, combined with the wide steady state distribution of MLKL protein throughout the human brain [37] supports the existence of important roles for MLKL in cell types essential for neurological function in humans. The collective global frequency of *MLKL* LOF alleles is 0.0007, (equivalent to 1–2 in every 1000 individuals being heterozygous carriers) (Figure 2B). The identification and clinical description of additional human individuals that are homozygous or compound heterozygous for *MLKL* LOF alleles will shed further light on whether the absence of MLKL alone is sufficient to give rise to neurodegenerative disease, or if other genetic or environmental events are indeed at play.

## An *MLKL* missense *hypo*morph in Maturity Onset Diabetes of the Young (MODY)

Recently, a heterozygous *MLKL* missense variant p.G316D (rs375490660) was reported to be associated with Maturity Onset Diabetes of the Young (MODY) in a Palestinian family [25]. This variant segregates with a known MODY-associated LOF gene variant in the gene encoding the pancreatic transcription factor PDX1, and other predicted deleterious variants in the genes *ERN2* (endoplasmic reticulum stress), *NIPAL4* (Mg^2+^ transporter activity) and *SPTBN4* (actin binding). When compared with exogenously produced MLKL^WT^, protein levels of exogenously produced *MLKL* p.G316D are significantly reduced. RIPK3-phosphorylated (Ser358) MLKL p.G316D are basically undetectable despite normal upstream signalling. Exogenous expression of *MLKL* p.G316D was also unable to reconstitute necroptotic cell death in two lineage-distinct CRISPR-Cas9 *MLKL^KO^*^/KO^ human cell lines and did not appear to influence the function of endogenously expressed MLKL^WT^ in *MLKL* wild-type cells (simulating the heterozygous state of patients). While the stability and function of endogenously expressed *MLKL* p.G316D were not verified in patient-derived cells in its endogenous heterozygous context, normal levels of *MLKL* p.G316D mRNA in patient derived cells indicates that this missense mutation does not lead to reduced mRNA stability [25]. For this reason, we have chosen to classify this missense variant for the purpose of this review as a ‘hypomorph’ as opposed to a full LOF variant, as the phospho-Ser358-independent functions of *MLKL* p.G316D are yet to be investigated.

With a global minor allele frequency of 1.062 × 10^−5^, *MLKL* p.G316D homozygotes have not been recorded in the gnomAD database. How a monoallelic reduction in necroptotic cell death contributes to the aetiology of diabetes in this family remains a matter of investigation. *Mlkl^KO/KO^* mice do not display any of the hallmarks of diabetes when fed a standard laboratory mouse chow diet, and actually demonstrate *improved* glucose and insulin tolerance relative to *Mlkl^Wt/Wt^* controls when placed on a high fat diet for more than 12 weeks [38,39]. Investigating whether *Mlkl* gene knockout or mutation is a genetic modifier in mouse models of spontaneous diabetes (e.g. *Pdx1^Wt/KO^* mice [40]) could provide a useful route for the mechanistic dissection of these human findings.

Importantly, such studies can also prompt examination of potential risk posed by other human *MLKL* LOF and missense variants to the incidence of MODY. With no ‘gold standard’ criterion or threshold for diagnosing MODY in human patients, broadening gene panels for precision genetic testing will inform clinical diagnosis and management [41].

## Common *MLKL* missense variants in inflammatory disease, historically important *hyper*morphs?

The biological consequences of spontaneous and rationally designed *MLKL* gain of function or *‘*activating’ mutations have been described both in vitro and in vivo in murine models [30,42,43]*.* Human MLKL however is less amenable to rationally designed modifications that confer RIPK3 independent activity [43,44]. In 2020, a random, chemically induced germline missense mutation in mouse *Mlkl* was shown to result in systemic lethal neonatal inflammation in homozygotes and hematopoietic dysfunction in heterozygotes [27]. This mutation, *Mlkl* p.D139V, confers constitutive cell death activity to MLKL, removing the requirement of upstream necroptotic signalling and RIPK3-mediated phosphorylation. The human equivalent, *MLKL* p.D140V, has been observed only once, and only in heterozygous form in humans in the gnomAD database (rs1330913532). Interestingly, three very closely situated *MLKL* missense gene variants are amongst the most prevalent *MLKL* single nucleotide polymorphisms (SNPs) present in humans. These SNPs, *MLKL* p.Ser132Pro (rs35589326, global MAF — 0.0138), p.Arg146Gln (rs34515646, global MAF — 0.0152) and p.Gly*202Val (rs144526386, global MAF — 0.0123, *non canonical transcript)- have not been previously associated with human disease in any previous genome-wide- or phenome-wide-association studies (GWAS or PheWAS) individually. They were however shown to occur *in trans* at 10–12 times the expected frequency in a cohort of patients suffering from chronic recurrent multifocal osteomyelitis (CRMO) [27]. While this association has not been independently replicated in a second CRMO patient cohort to date, this finding does fit nicely with mouse and human studies that implicate ‘unchecked’ necroptosis in the progression of inflammatory diseases [20,26].

An analysis of the cellular function of the protein products of these common human *MLKL* SNPs relative to wild-type MLKL is yet to be reported in the literature. However, the hypermorphic nature of the closely situated mouse mutation *Mlkl* p.D139V does raise important questions as to the evolutionary significance of such high allele frequencies. An estimated 8% of the global population are heterozygous carriers of *MLKL* p.S132P, p.R146Q or p.G*202V based on gnomAD data, and 167 are homozygotes. Many reported hypermorphic variants in immune-related genes that confer a survival advantage to specific pathogens have achieved high allele frequency in certain populations [45,46]. Many such SNPs are also associated with an increased relative risk of autoinflammatory diseases like inflammatory bowel disease and systemic lupus erythematosus [46]. The identification of such evolutionary triads (pathogen - gene hypermorph — inflammatory disease) has the potential to blaze important trails into the development of novel anti-microbials and immunomodulatory therapies alike.

By this same evolutionary logic, *MLKL's* implication in diseases like diabetes and Alzheimer's also warrants the investigation of any historical survival advantage conferred by *MLKL* gene variants or expression Quantitative Trait Loci (eQTL) with regards to nutrient acquisition and storage. An intriguing example of this was described recently for MLKL's upstream activator RIPK1. Human gene variants that increase *RIPK1* gene expression were found to be associated with obesity [47]. Nutrition and metabolism serve alongside pathogens as major evolutionary drivers of human genetic variation, and are core determinants of the common disorders (e.g. cardiovascular disease), that contribute substantially to disease burden in the modern world [48].

## *MLKL* gene variants as modifiers of complex polygenic human traits and diseases

To date, *MLKL* gene variants have only been weakly associated with human traits or changes in disease risk in conventional GWAS. The highest '*P*' value reported for an *MLKL* variant in a published GWAS was for a non-coding SNP rs2057805 (global MAF 0.2489) located in an upstream regulatory region in adolescent idiopathic scoliosis [49]. This association reached genome wide significance with a Bonferroni-adjusted *P*-value of 3 × 10^−9^. Other notable associations of *MLKL* SNPs with quantitative human traits that are significant or approaching genome wide significance include one with comparative height at age 10 (*P* = 5.26 × 10^−7^) [50] and another with mean reticulocyte volume, *P* = 1 × 10^−8^ (Global Biobank Engine, Stanford, CA (URL: http://gbe.stanford.edu accessed September 2021)) [22]. Interestingly, three non-coding SNPs in MLKL's obligate activating kinase RIPK3 are each very strongly associated with standing height in humans, *P* values <4 × 10^−23^ [22]. While the small ‘beta’ log odds ratios for each of these *MLKL* and *RIPK3* SNPs described indicate that they account for only very low proportions of the phenotypic variance in these quantitative traits, they nonetheless highlight the potential of such unbiased approaches for uncovering unexpected gene-function links [51].

## Disease causing gene variants identified in important upstream regulators of MLKL

When considering the pathophysiological impacts of necroptosis, we must not only consider genetic variation in *MLKL* itself*,* but also variation in genes that can inappropriately unleash, amplify, or even dampen the cellular activity of MLKL. Based on observations of important MLKL regulatory genes gleaned from genetically modified mice [20], we have plotted for the purposes of this review the number of disease associated human mutations reported in ClinVar [52], as accessed in September 2021 (Figure 2A). This tally is strongly dominated by *RIPK1*, *TNFAIP3* (encoding A20) and *CASP8*, where several unique LOF or missense mutations are characterised by autoinflammatory and lymphoproliferative syndromes [53]. It is important to note however, that the identification of pathogenic gene variants is heavily biased towards those that associated with severe or atypical disease. A higher number of pathogenic *RIPK1, TNFAIP3* and *CASP8* mutations in ClinVar does not equate to a larger contribution to the heritable variation in disease risk, when considering all disease burden in the human population. In fact, it is likely quite the opposite. Examining the mutational spectrum of ∼141 000 individuals catalogued by the gnomAD database permits a simple visualisation of the high evolutionary constraint on LOF variation in *RIPK1*, *TNFAIP3* and *CASP8* [54] (Figure 2B). *MLKL* and *ZBP1* each exhibit a higher summed frequency of predicted LOF alleles than *RIPK1*, *TNFAIP3* and *CASP8* combined, and both are dotted with a larger repertoire of more common (>MAF 0.001) missense gene variants (Figure 2C). The higher missense and LOF variation tolerance profiles of *MLKL, RIPK3 and ZBP1* indicate that gene variants are less likely to result in a loss of reproductive fitness (e.g. severe disease in children and young adults) that would cause them to be subject to purifying selection over time [55]. By this same logic however, the higher levels of standing protein-coding variation in *MLKL, RIPK3 and ZBP1* genes also increases the statistical likelihood that these genes can act as low effect size modifiers of polygenic traits in humans (e.g. disease risk, infection response, height or weight) [56].

## *MLKL* and the varied manifestations of inborn errors in *CASP8*

A full decade before MLKL's role in cell death was discovered, inherited deficiency of Caspase 8 was reported to mediate autoimmune lymphoproliferative syndrome (ALPS). Chun et al. [57] reported homozygosity of a *CASP8* variant (p.R248W) in two siblings that presented with lymphadenopathy and splenomegaly. Caspase-8 p.R248W exhibited reduced stability and resulted in an enzymatically inactive protein, with peripheral blood lymphocytes defective in CD95-induced apoptosis. Caspase-8 activity is an important determinant of programmed cell death downstream of death receptor and inflammasome activation [58]. The absence or inhibition of Caspase-8 or c-FLIP_L_ activity facilitates the stabilisation of RIPK1 and RIPK3 [59–62] (both shown to be cleaved by Caspase-8 and/or Caspase-8-c-FLIP_L_ heterodimers). Amongst other signalling events, this also promotes the formation of the necrosome and MLKL-mediated cell death. Mice deficient in Caspase 8 die at embryonic day 10.5 but *Casp8^WT/K^*^O^ heterozygotes are phenotypically normal [63]. *Casp8^KO/K^*^O^ embryonic lethality does not occur on a *Mlkl^KO/KO^* background, but *Casp8^KO/K^*^O^, *Mlkl^KO/KO^*mice do go on to develop progressive lymphadenopathy [64]. In light of this later observation, dysregulated MLKL function and necroptotic cell death is likely to be more pertinent to the many examples of human *CASP8* variant -borne disease that do not manifest as ALPS.

Caspase-8 deficiency manifests as very early-onset Inflammatory Bowel Disease (VEO-IBD) in some individuals. Lehle et al. [65] reported three unrelated patients with homozygous *CASP8* mutations (p.Q237R and p.R265W (equivalent position to canonical isoform R248W)) that presented with diarrheal, perianal disease, failure to thrive and discontinuous severe proctocolitis, as well as increased susceptibility to bacterial and viral infections. Primary patient cells and cells engineered to express *CASP8* p.Q237R exogenously exhibited increased IL-1β release in response to lipopolysaccharide (LPS) priming, that could be blocked by NLRP3-mediated inflammasome inhibitor MCC950 and the MLKL inhibitor necrosulfonamide. Furthermore, *CASP8* p.Q237R, but not p.R265W, was also shown to predispose engineered HT-29 colon carcinoma cells to increased MLKL oligomerisation and death when exposed to a necroptotic stimulus [65]. The important role of *MLKL* in this disease scenario is further supported by recent observations that *MLKL* fully mediates the ileitis and colitis observed in mice with intestinal epithelial cell-specific deficiency of Caspase-8 [66]. Chun et al.’s [65] and Lehle et al.’s [67] clinical descriptions of distinct disease manifestation in individuals harbouring *CASP8* p.R248W/R265W also highlights that unchecked necroptosis may be variably expressed as a phenotype of the same *CASP8* mutation, depending on the individual . The genetic or environmental determinants of this disparate role of *MLKL* remains an important line of future investigation into the potential toxicity of Caspase-8 targeted drugs.

## Choose your battle: MLKL and disease associated with inborn errors of *RIPK1*

Given the important position of RIPK1 at the nexus of several cellular signalling pathways, MLKL and necroptosis account for a significant portion, but not *all* of, the downstream effects born of genetically encoded RIPK1 dysfunction. In 2018, Cuchet-Lourenco et al. [68] reported four individuals that were homozygous for three unique *RIPK1* LOF nucleotide deletions, and a number of additional patients with homozygous *RIPK1* LOF alleles were reported soon after [69]. Patients presented with early-onset inflammatory bowel disease, lymphopenia and arthritis of varying severity. One patient also suffered from growth restriction, severe motor delay and mild intellectual disability [70]. An increased susceptibility to viral, bacterial and fungal infection was also reported [53,71]. Patient fibroblasts isolated from one of these patients were shown to be more susceptible to cell death in the presence of TNF and polyI:C. These cells were protected from death by the MLKL inhibitor necrosulfonamide, strongly implicating necroptosis [68]. Similarly to *Casp8*, *Ripk1* knockout in murine models is homozygous lethal, with mice dying during the immediate post-natal period due to systemic inflammation and cell death in multiple tissues. This inflammation is ameliorated in a *Mlkl^KO/KO^* background, but mouse pup survival is only extended by a few days [72]. The severe phenotype of *RIPK1^ KO/KO^* mice is only bypassed when both necroptosis *and* extrinsic apoptosis are removed from the equation by compound genetic crosses [72–74]. Homozygous *RIPK1* LOF in humans can lead to severe disease, but is not incompatible with life as it is in mice. Is this difference intrinsic to inter-species differences in RIPK1 itself, or can it be explained by key species-specific differences in the regulation of downstream signal effectors like MLKL?

Compounding these questions are disease causing missense *RIPK1* gene variants that prevent RIPK1 cleavage by caspases. Heterozygosity of such ‘cleavage resistant’ alleles is sufficient to induce a severe periodic inflammatory syndrome in humans characterised by fever and lymphadenopathy [75–77]. Homozygosity reduces the lifespan of mouse embryos to E10.5, well shorter than that observed for *Ripk1^KO/KO^* mice and for reasons that cannot be remedied by the deletion of *Mlkl* [76,78]. The hyperinflammatory phenotype of heterozygous adult mice is similarly unaltered by the genetic deletion of *Mlkl* (N. Lalaoui, unpublished).

## *RIPK3* variants in human health and disease

To date, there have been no clinical descriptions of humans that are homozygous for *RIPK3* LOF gene variants. One individual that is homozygous for a predicted LOF *RIPK3* allele was recorded as part of the ‘human knockout project’, though details of this individual's phenotype were not provided [79]. The allele in question, *RIPK3* p.Pro493ThrfsTer9 (rs531266348), contains a frame shift insertion that may lead to a short C terminal truncation as opposed to a full LOF. As such, its classification as ‘Loss of Function’ is classified as low confidence in the gnomAD database, as is the only other potential LOF variant found in homozygous form; *RIPK3* p.Arg422Ter (rs146886719). Similar to the *Mlkl^KO/KO^* mouse, *Ripk3^KO/KO^* mice do not show any overt signs of spontaneous neurodegenerative disease or diabetes under steady state conditions, even when aged beyond 12 months [80,81]. Interestingly, *Ripk3^ KO/KO^* mice do show evidence of disturbed bone architecture and increased osteoclasts, supporting the reported association of the RIPK3 *cis*-eQTL rs3212240 with lower estimated bone mineral density in a human cohort [82]. Given our discussion of gene variation and evolutionary fitness, it would be remiss not to mention reports that *Mlkl^KO/KO^* and *Ripk3^KO/KO^* mice show a significantly reduced propensity for reproductive organ aging [81,83]. Necroptosis deficient mice sire more pups at advanced age than their wild-type counterparts. This advantage is offset by the reduced fitness of the pups born to older fathers, but certainly warrants consideration of the influence of necroptosis gene variation and necroptosis drugs in human fertility going forward [81].

## Conclusion

This review gives an overview of the current landscape of *MLKL's* role in heritable disease. Two distinct, *de novo MLKL* LOF variants have been shown to segregate with a severe familial neurodegenerative disorder and familial maturity onset diabetes of the young (MODY). While *MLKL* LOF alleles are more prevalent in the population relative to other key genes involved in programmed cell death, they are dwarfed in frequency (>100-fold less frequent) by a series of common non-conservative protein modifying mutations that cluster at key functional domains of MLKL. The identification and clinical description of additional families and individuals carrying homozygous and heterozygous *MLKL* LOF gene variants is not unlikely given the growing uptake of whole genome sequencing for clinical diagnosis. While rare, these individuals will reveal new and important insights into the role of *MLKL* and disease aetiology and the basic molecular mechanism of MLKL's varied cellular functions. They will help to focus and fine-tune the study of the trait- and disease- modifying potential of the *MLKL* protein coding variants carried by >10% of the global population, unlocking new clinical indications and contraindications for drugs that target necroptosis and its important upstream regulators.

## Perspectives

- Naturally occurring human gene variants are important tools for the study of necroptosis at the molecular and pathophysiological level.
- Rare and *de novo* necroptosis gene variants have been implicated in autoinflammatory disease, neurodegeneration and metabolic disease.
- More than 10% of individuals carry protein-coding single nucleotide polymorphisms in *MLKL*. Studying their role in human health and disease will uncover new clinical indications and contraindications for drugs that target necroptosis and its important upstream regulators.

## Abbreviations

ALPS: autoimmune lymphoproliferative syndrome
CRMO: chronic recurrent multifocal osteomyelitis
GWAS: Genome Wide Association Studies
LOF: loss of function
MLKL: mixed lineage kinase domain-like
MODY: Maturity Onset Diabetes of the Young
RIPK1: receptor interacting protein kinase 1
SNPs: single nucleotide polymorphisms
TNFR1: tumour necrosis factor receptor 1
ZBP1: Z-DNA Binding Protein-1
